# Deep Learning in the Ubiquitous Human–Computer Interactive 6G Era: Applications, Principles and Prospects

**DOI:** 10.3390/biomimetics8040343

**Published:** 2023-08-02

**Authors:** Chunlei Chen, Huixiang Zhang, Jinkui Hou, Yonghui Zhang, Huihui Zhang, Jiangyan Dai, Shunpeng Pang, Chengduan Wang

**Affiliations:** 1 School of Computer Engineering, Weifang University, Weifang 261061, China; chunlei.chen@wfu.edu.cn (C.C.); houjk@wfu.edu.cn (J.H.); zyh@wfu.edu.cn (Y.Z.); huihui@wfu.edu.cn (H.Z.); daijy@wfu.edu.cn (J.D.); pangshp@wfu.edu.cn (S.P.); 2 School of Cyberspace Security, Northwestern Polytechnical University, Xi’an 710072, China; zhanghuixiang@nwpu.edu.cn

**Keywords:** human-centric, 6G, deep learning

## Abstract

With the rapid development of enabling technologies like VR and AR, we human beings are on the threshold of the ubiquitous human-centric intelligence era. 6G is believed to be an indispensable cornerstone for efficient interaction between humans and computers in this promising vision. 6G is supposed to boost many human-centric applications due to its unprecedented performance improvements compared to 5G and before. However, challenges are still to be addressed, including but not limited to the following six aspects: Terahertz and millimeter-wave communication, low latency and high reliability, energy efficiency, security, efficient edge computing and heterogeneity of services. It is a daunting job to fit traditional analytical methods into these problems due to the complex architecture and highly dynamic features of ubiquitous interactive 6G systems. Fortunately, deep learning can circumvent the interpretability issue and train tremendous neural network parameters, which build mapping relationships from neural network input (status and specific requirements of a 6G application) to neural network output (settings to satisfy the requirements). Deep learning methods can be an efficient alternative to traditional analytical methods or even conquer unresolvable predicaments of analytical methods. We review representative deep learning solutions to the aforementioned six aspects separately and focus on the principles of fitting a deep learning method into specific 6G issues. Based on this review, our main contributions are highlighted as follows. (i) We investigate the representative works in a systematic view and find out some important issues like the vital role of deep reinforcement learning in the 6G context. (ii) We point out solutions to the lack of training data in 6G communication context. (iii) We reveal the relationship between traditional analytical methods and deep learning, in terms of 6G applications. (iv) We identify some frequently used efficient techniques in deep-learning-based 6G solutions. Finally, we point out open problems and future directions.

## 1. Introduction

### 1.1. Background

The unparalleled burst of next-generation communication technologies opens a new vision for human–computer interaction. Novel human–computer interaction technologies, like remote virtual reality, distant augmented reality and metaverse, all put forward high demand on the underlying communication infrastructure.

Nowadays, the rapid growth of 5G technologies has shown an identified prospect that 5G is being irreversibly standardized and commercialized. Although 5G can support higher-quality service than 4G, no pathbreaking technology accounts for this improvement. In detail, 5G follows the basic mechanism of performance improvement, which relies on more consumption of spectral and hardware resources. The increasing application of 5G is generating novel communication abilities and services that are supposed to cause innovation in society. It is obvious that the envisioned society innovation will induce challenges that are beyond 5G. The next-generation communication technology will be a cornerstone for the future physical-cyber-fused world and meet the communication requirements of humans and computers.

As a result, academic and industrial communities have opened the way for the next-generation 6G communication networks. The 6G technology should facilitate a physical-cyber continuum, which seamlessly integrates the physical and digital worlds. This continuum should fuse humans and computers (including the embedded computers in other devices) and thus enable seamless and immersive interaction.

In our work, we follow the description of “human–computer interaction” as below. The importance of computers or computer-embedded devices is constantly rising in the last decades. Moreover, the human–computer interaction research mainly focuses on the evolution of the way of interaction. Nowadays, we are entering the ubiquitous computing age. An increasing number of human–computer interfaces are penetrating into every corner of human society. There is a significant shift in the role of computers, i.e., computers are not only performance-oriented but also becoming an imperceptible part of daily life. The dramatic advances of various digital sensors let the interfaces go far beyond traditional command line, keyboard and mouse. The interaction between human and computers are becoming ubiquitous yet increasingly invisible. Due to the huge data amount constantly generated by enormous digital sensors and the undoubted requirement of interconnecting massive computers, 6G is an indispensable backbone to facilitate the interaction by providing underlying infrastructures.

It is argued that conventional mobile communication remains the most dramatic deployment scenario despite the rise of other applications in both ubiquity and scale [1]. Therefore, 6G should be human-centric, which highlights the features of security, secret and privacy [2,3,4]. Moreover, 6G is supposed to be equipped with enhanced coverage, transmission rate and inter-user connectivity anywhere [5].

### 1.2. Related Works

There have been some reviews or surveys on interactive 6G applications or relevant topics. Lu et al. point out the application context, technologies and prospects of 6G, with the purpose of identifying frontier issues for further research [6]. The work of [7] reviews the machine-learning methods that can be applied to 6G wireless networks. However, this work mainly focuses on the principles of these methods and paid less attention to machine-learning-enabled practical 6G applications. Salh et al. review the deep-learning-enabled solutions to 6G ultra-reliability and low latency communications and suggest more 6G-URLLC-oriented research on deep learning [8]. Since healthcare or medical applications account for an important context of the future 6G-enabled interaction, Jeong et al. point out four typical medical issues that can be elegantly addressed by FL (federal learning). They also propose a general roadmap for applying and investigating key problems in fitting FL into such applications [9]. Mucchi et al. review the signal processing technologies for 6G communications. They point out that machine learning methods play an indispensable role in 6G signal processing. However, most of the existing machine learning methods stem from other fields like image recognition and natural language processing. As a result, many technical issues are to be addressed before machine learning efficiently fits into the 6G applications [10].

Machine learning methods can be categorized into two types: traditional analytical methods and data-driven methods. The former relies on rigorous mathematical reasoning (like Integer Linear Programming). The latter trains inferencing models with massive data, and currently the mainstream method is deep learning. Despite that, the analytical methods can be formally proven and thus more rigorous, deep learning is superior when the rigorous mathematical reasoning is excessively complex or time-consuming.

Deep learning has dramatic advantages over traditional analytical machine learning methods [11,12]. First, deep learning can obtain better performance if the data volume is huge. In contrast, traditional machine learning cannot fully benefit from the constantly accumulating massive data. Second, deep learning depends less on feature engineering. Deep learning can automatically extract features in a layer-wise mode to represent the complex high-dimensional input data using a nested hierarchy of features. However, traditional machine learning typically requires manually extracted features by domain experts. This requirement may be excessively harsh due to the unprecedented complexity and dynamic nature of the 6G communication networks. Third, deep learning can surpass traditional methods with regard to human–computer interaction applications, like human behavior understanding, speech recognition, computer vision, etc. The 6G communication network is widely believed to be an indispensable intermedia for next-generation human–computer interaction. Deep learning is a promising solution to boost human–computer interaction applications in the envisioned 6G era [13,14,15,16,17,18].

### 1.3. Main Contributions and Outline

Due to the high complexity of 6G communication networks, it is a challenging task to bridge the gap between deep learning and 6G. Potential challenges can stem from the complexity of the underlying hardware [19,20], novel characteristics of the carrier-waves [21,22], high dynamic features of the channel [23,24], heterogeneity of the application requirements [25], tradeoffs among diverse performance metrics [26], lack of training data [27], etc.

In view of the aforementioned challenges, we investigate representative research works. We identify some key issues and clarify the roadmap. The main contributions are summarized as follows.

First, for the systematic view: we investigate representative deep-learning-based 6G solutions from a systematic perspective. Concretely, we assume a three-layer architecture of 6G technologies and research the representative works within this architecture. As a result, we find out some common issues like the significant role of deep reinforcement learning in the 6G context.

Second, for the source of training data: we investigate solutions to the lack of training data for 6G communications. Many deep learning methods rely on abundant training data to train a huge number of neural network parameters and thus achieve large capacity and sufficient generalization ability of the neural network. However, 6G communication is still in its infancy. It is currently difficult to obtain training data from 6G communication networks. We investigated several ways to conquer this predicament.

Third, for the relationship between analytical methods and deep learning: we find that deep learning can be an alternative to analytical methods or even cooperate with analytical methods. On one hand, deep learning can cope with 6G problems when the analytical methods are NP-hard. On the other hand, deep learning can achieve acceptable accuracy with dramatically lower complexity, even if the analytical counterpart is resolvable in polynomial time.

Fourth, for the frequently used techniques: we investigate some representative and efficient techniques in deep-learning-based 6G solutions. (i) The deep-learning-based multi-objective optimization can efficiently tradeoff among multiple performance metrics. (ii) The decision space of deep-reinforcement learning can be simplified to accelerate the training. (iii) Distributed deep learning can boost ubiquitous intelligence in the 6G context.

Moreover, we believe that the existing deep-learning-based 6G solutions open a promising way to the future 6G-enabled ubiquitous interaction. But many issues are to be addressed, including novel materials of the underlying hardware, cross-layer design of deep learning solutions, etc.

The rest of this paper is organized as follows. Section 2 discusses the representative applications of the envisioned interactive 6G era. Section 3 reviews the representative research works that fit deep learning into the 6G context. Section 4 discusses some vital issues and proposes our main contributions. Section 5 highlights the open problems. Section 6 concludes our work and points out future works.

## 2. Applications and Challenges of the Envisioned 6G Context

### 2.1. Typical Application Scenarios

**Figure 1:**
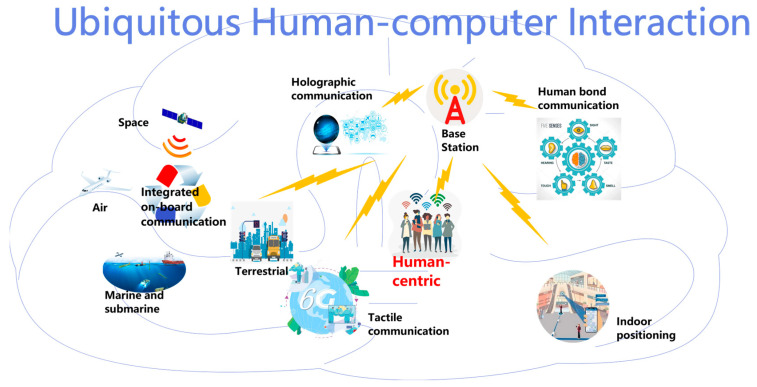
Ubiquitous human–computer interaction.

In view of the future human–computer interaction prospects, 6G is supposed to play vital roles in certain application scenarios, including but not limited to the following (Figure 1).

1. Next-generation mobile communications. Owing to the vision of ubiquitous human–computer interaction, mobile communications still occupy the dominant position in 6G and conventional cellular phones will continue to play a pivotal role. Challenges may come from several aspects: First, raising the network coverage in a prompt and cost-effective manner. Second, maintaining a super-speed data rate and lower transmission latency with highly reliable connections. Third, expanding the battery duration of mobile devices. Fourth, decreasing the cost of mobile communication. Fifth, accomplishing a systematic design to integrate features of security, secret and privacy, as well as effectively addressing the aforementioned challenges.
2. Holographic communications. 6G is expected to convert traditional video conferences into virtual immersive interactive meetings, which need to transmit holographic data with negligible latency. In this scenario, an unprecedentedly large bandwidth is a necessity due to the fact that three-dimensional images and stereo audio need to be conveyed and reconfigured as required.
3. Tactile Communication. In addition to the visual and auditive data, haptic data can further enhance the interactivity of virtual immersive applications. Tactile communication can capacitate the real-time remote exchange of haptic data. Typical applications cover healthcare, teleoperation and collaborative automatic driving, where the 6G-enabled tactile communication network can be a high-speed channel as part of the control loop. For example, healthcare applications like remote robot surgeries rely heavily on 6G-powered IoT (Internet of Things). Diversified network nodes and devices are integrated to accommodate healthcare applications, which propose high demand on reliability and latency of the communication network [28]. Meticulous cross-layer orchestration should be performed to reshape the communication network such that the strict constraints of these applications can be satisfied. For instance, novel physical layer solutions should be proposed to redesign the underlying circuits of the communication system. All delay sources should be elaborately reviewed throughout the communication protocol stack.
4. Human bond communications. Since 6G is supposed to be the cornerstone of human-centric communications, the concept of human bond communications is to enable users to interact with devices with the human five senses or even breathing. Consequently, the devices may recognize human bio-profile and biological features in a remote manner. Moreover, hybrid communication technologies are required to not only identify and replicate human biological features but also convert the features into various physical signals and transmit them.
5. Efficient indoor positioning. Nowadays, outdoor positioning is proven to be full-blown and accurate in common application scenarios. Nevertheless, indoor positioning is yet in its infancy, due to the complicated indoor electromagnetic environment [29,30]. Precise and robust indoor positioning technologies will thoroughly reinvent the living habit of mobile users and create new increasing spots of economic boom. Additionally, the academic and industrial communities are reaching a consensus that mere RF (Radio Frequency) communication technologies cannot meet the demands put forwards by indoor positioning. By contrast, novel non-RF communication technologies are already in the vision of the 6G era.
6. High-quality on-board communication. Despite the significant success of 4G and 5G, on-board communication is still a challenging topic. The quality of onboard communication is hampered by enormous factors, including high-speed motion, Doppler shift, frequency handover, insufficient coverage, etc. Satellite communications endow onboard communication with an acceptable quality of service, yet the cost is excessively high, especially under in-flight environments. High-quality on-board communication requires both innovative communication technologies and brand-new network architectures.

### 2.2. Arising Challenges

**Figure 2:**
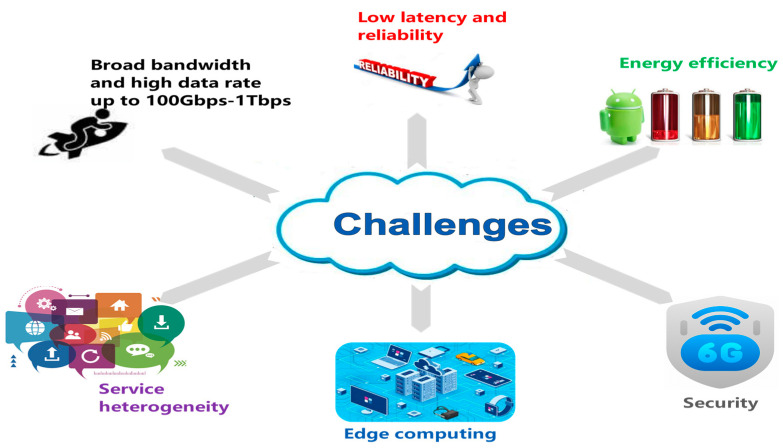
Challenges to ubiquitous human–computer interaction.

Due to the novel demands proposed by the aforementioned applications, 6G may need to conquer the challenges regarding the following application scenarios (Figure 2).

Although we outline the challenging issues and discuss them separately, each of the issues requires holistic design in a future real 6G application. Take low latency as an example. On the hardware signal source level, we need new devices to provide mmWave (millimeter wave) and THz (terahertz) communications [31]. On the channel level, emerging technologies like IRS (intelligent reflection surface) [32] are required to ensure spectrum efficiency and the favorable propagation environment. Moreover, on the network architecture level, edge computing is an indispensable mechanism to achieve load balance, effective data caching and less network congestion. In addition, SDN and NFV play an imperative role on the application level to guarantee low latency for diverse applications. As a result, every issue requires a systematic scheme other than a single technique.

#### 2.2.1. Broad Bandwidth and High Transmission Rate

1. Limitations on the traditional carrier wave technologies. In the past decades, research works on mobile communications mainly concentrated on the microwave band or an even narrower band, which is dedicated to boosting spectrum efficiency using a relatively low frequency. In this era, representative solutions include CDMA (code-division multiple access), OFDM (orthogonal frequency-division multiplexing), multi-level modulation, etc. On one hand, such solutions have raised spectrum efficiency; on the other hand, performance improvement is increasingly impeded by physical limits.
2. mmWave and THz communication: advantages and challenges. Adopting broader bandwidth becomes an inevitable routine to further increase the data rate and capacity. Higher frequency spectra like mmWave and THz wave band will enable unprecedentedly broader bandwidth for 6G [19,20]. However, mmWave and THz wave are facing unneglectable challenges like signal attenuation and RF chips.

Millimeter-wavelength or THz-band radio waves dramatically tend to propagate along a straight line. Moreover, such waves are significantly attenuated by shields, such as vehicles, buildings, animals, etc. Therefore, it is a challenging task to maintain and secure the line-of-sight (LOS) context between the transmitter (TX) and receiver (RX) in mobile communications. Consequently, this challenge necessitates the in-depth analysis of the radio propagation features and develops the propagation model to precisely simulate the propagation. 

In terms of mm-wave and THz wave, it is a great challenge to design and manufacture RF (radio frequency) chips [33,34], including oscillators, filters, etc. Such chips put forward high requirements for low power consumption and efficient thermal design. Additionally, optical technologies may play an indispensable role in frequency generation, conversion and control of THz waves [35,36]. Design and manufacture of high-frequency communication circuits and antennas are confronted with significant barriers due to the fact that high-frequency results in high wiring loss. Moreover, beamforming and scheduling is also a challenging task [22]. And, it is even more challenging in high-speed mobility scenarios [21].

As aforementioned, both the propagation model and RF chips need careful investigation. Nevertheless, the design spaces of the two topics are high-dimensional and excessively large, which means conventional optimization methods may be insufficient to resolve the problems.

The representative supporting technologies of mmWave and THz band communication include beam forming, IRS [37], etc. Although the conventional analytical optimization methods can reach a reasonable solution, unneglectable obstacles still hinder the practical application due to but not limited to the following reasons. First, the conventional methods result in high complexity of hardware under mmWave and THz environment. Second, high power consumption is unavoidable with regards to the conventional methods in mmWave and THz context. Third, conventional analytical inferencing typically induces unacceptable time overhead if applied to mmWave and THz problems.

#### 2.2.2. Latency and Reliability

1. The perspective of carrier wave and coding. Resource scheduling costs account for unneglectable latency in wireless communication. GF (Grant-Free) resource allocation [38] has been proposed by 3GPP (3rd Generation Partnership Project) [39], which is dedicated to accommodating low latency transmission in both uplink and downlink. In terms of uplink, it is a problem of CG (Configured Grant); with regards to downlink, it is Semi-Persistent Scheduling. In CG, UE (User Equipment) sends data through PUSCH (Physical Uplink Shared Channel) resources with the absence of requesting from the gNB (gNodeB). The PUSCH resources are configured and assigned to UEs in advance via downlink control messages. A downlink control message can be a DCI (Downlink Control Signal) or RRC (Radio Resource Control) signaling. This circumvents signal exchanging, like SR (Scheduling Request) or BSR (Buffer Status Report). Such signal exchanging can otherwise induce higher latency. In the era of 5G, the 2-step RACH (Random Access Channel) procedure is introduced to transmit data over shared resources with a higher data rate [40]. Nevertheless, this method merely works in initial access and is not available for data transmission during the connected mode. Alternative representative methods include a repetitive transmission [41], NOMA (Non-Orthogonal Multiple Access) [42,43] and advanced receivers [44]. The former two methods can achieve acceptable performance when network traffic is low yet face dramatic performance degradation under intensive traffic. Despite the advances, receivers are confronted with increased hardware complexity and power consumption hinder its wide application.
2. Propagation environment. In order to achieve URLLC for eMBB (enhanced Mobile Broadband) and mMTC (massive Machine-Type Communication), spectrum efficiency are indispensable issues. However, the tradeoff among reliability, latency and spectrum efficiency is a challenging task. Enormous researchers have proposed solutions to address the challenge. For instance, new air interface accesses like cell-free with massive multiple-input multiple-output, non-orthogonal multiple accesses, and rate-splitting multiple accesses. These solutions mainly focus on the transmitter and/or receiver side [23]. Nevertheless, the performance improvement may be limited by the propagation environment. IRS is a recently emerged solution to address this issue. IRS can adapt to the propagation environment in a cost-effective and energy-efficient way. Whereas, it is not a trivial job to integrate IRS into the wireless network architecture. Moreover, the design and optimization of the IRS is a daunting job due to the high complexity [24].
3. NFV-level tradeoff. Challenges to latency issues come from not only the radio-wave level but also the application level [45]. NFV (Network Function Virtualization) is a promising technology in the emerging 6G era [46]. NFV can leverage general-purpose hardware to virtualize network functions like routers and firewalls, which conventionally rely on dedicated hardware. With the growing adoption of URLLA (Ultra-Reliable Low Latency Applications) in the next-generation wireless networks, reliability and latency are increasingly important in a NFV context. However, efforts on optimizing reliability and latency typically face a dilemma, due to the fact that the two-performance metrics are somewhat in opposition to each other. Although the optimization can be formulated as an integer linear programming problem [47,48,49], the solving process is excessively time-consuming.

#### 2.2.3. Energy Efficiency

1. Energy-demanding features of 6G networks. Power consumption and battery duration are common issues in wireless communication networks. Whereas, 6G is confronted with some new challenges, including but not limited to the following aspects [50,51,52].

First, network infrastructure. The maximum transmission distances of THz wave and mmWave are typically limited to ten meters and one hundred meters, respectively. Therefore, the coverage area of a forthcoming THz BS (Base Station) is approximately as small as one hundred square meters, which means a dramatically increased amount of BSs. Second, massive data migration and processing. The computation and user-oriented services will increasingly migrate from local devices to the cloud and edge servers for further processing, which also accounts for an unneglectable part of the power consumption. Third, AI technologies are broadly adopted for autonomous network control, adaptive data transmission and application service customization. Consequently, diverse AI programs will penetrate into every node of the 6G networks. While an AI program typically runs enormous floating-point operations that induce high power consumption of the processor.

2. The tradeoff between energy efficiency and other performance metrics. Low power dissipation and long battery duration are two research priorities in 6G. However, optimizing the power consumption of 6G devices is a daunting task due to the fact that power consumption and other performance metrics may have negative effects on one another [26]. For example, the tradeoff between power efficiency and spectral efficiency is probably an eternal problem in wireless communication, including 6G.

Another challenging tradeoff is between power efficiency and security. The barriers comprise but are not limited to, the following [53]. First, the heterogeneous nature of the 6G network. The 6G network intrinsically integrates tremendous devices and BSs with various functionalities under diversified working contexts. Such high integration leads to a variety of power consumptions and security requirements. It is challenging to adaptively cater to these heterogeneous demands, in terms of both power efficiency and security. Second, time variation. This nature makes the tradeoff even more difficult. Network conditions can be in constant change. The power supply of BSs may fluctuate, especially when they adopt renewable energy like solar or wind power. And, the moment when a security alert is triggered is somewhat stochastic. Consequently, the power scheme should be constantly tuned to tackle the complex time-varying demands.

#### 2.2.4. Security

6G security and privacy problems demand cross-layer considerations, which cover physical (hardware), network information (carrier-wave) and application layers.

1. Physical layers. A sheer number of research works propose to integrate newly emerged underlying technologies like THz communication, VLC (Visible Light Communication) and quantum computing to address security and privacy concerns. Such integrations are rooted in the physical layer. Nevertheless, some new threats arise due to the physical features of the new technologies [54]. In the frequency band of THz, the signal exhibits high directionality and enables LoS (Line of Sight) transmission. Unfortunately, an eavesdropper can set an object in the transmission path to scatter the radio wave to him [55]. Thus, THz communication is vulnerable to access control attacks and data theft. In addition to THz communication, VLC is also a promising support technology of 6G due to its advantages such as resistance to interference, high availability of spectrum and high transmission speed. However, VLC is also prone to eavesdropping due to its broadcasting nature and inherent LoS propagation.
2. Network information layers. In terms of the network information layer, tremendous novel network-layer technologies are already adopted in 5G. Such technologies are widely believed to still play indispensable roles in the envisioned 6G. Representative technologies include NFV, SDN (Software-Defined Networking), cloud computing, etc. However, SDN may be confronted with threats such as exposure of sensitive APIs (Application Programming Interfaces) to unauthorized software and DoS (Denial of Service) attacks [56]. Another instance is source location exposure. The envisioned physical-cyber continuum will open the way to social IoT, which involves the entire human society and facilitates the continuum with novel services [57]. However, such services may allow the acquisition of ubiquitous data flows and induce the risk of source location exposure. Such location exposure of assets or other targets may result in vulnerability to network attacks.

The work of [58] investigates countermeasures to source location exposure under 6G edge computing, using DRL. The authors take into consideration privacy loss, energy consumption and latency and then define an evaluation indicator. Subsequently, privacy loss is quantified as the probability of a successful attack based on prior knowledge. Finally, the task offloading problem of edge computing is formalized as a Markov decision process and is resolved by DRL with low privacy loss.

3. Application layer. On the application layer, the 6G applications typically collect and transmit sensitive and crucial data. Undoubtedly, the security and privacy issues of the forthcoming 6G networks should be tackled.

6G is mainly motivated by the vision of interconnected intelligence and AI-enabled networks [10]. Nevertheless, the union of AI and 6G is double-edged. On one hand, AI can significantly boost the performance of 6G networks. On the other hand, AI could also open a way for malicious or unauthorized operations. The ubiquitous adoption of AI in 6G may result in security issues under some scenarios, including but not limited to the following. First, trustworthiness: since the key functions can be controlled by AI, the trustworthiness of AI may be problematic, especially under some crucial application scenarios. Second, interpretability: interpretability is vital to guarantee controllability and accountability. The processes of AI-based operations should be readily unambiguous and visible to human experts or supervisory programs. Third, elasticity and feasibility: pervasive intelligence typically means distributed deployment of AI. While the latter may induce frequent network transmission and heavy network traffic, it may induce heavy computation and transmission costs to incorporate AI-enabled security schemes into the traffic [59].

#### 2.2.5. Edge Computing

The last decade witnessed the dramatic success of cloud computing in the scenarios of massive data storage and processing. In the cloud computing context, the remote data transmission between end nodes/edge servers and the cloud induces high bandwidth consumption, unneglectable latency and security threats. Meanwhile, the exponential rise of intelligent devices in 6G endows the edge with abundant distributed computational resources. Edge computing migrates part of computing and storage to the edge nodes (i.e., as close to the data source as possible), decreasing bandwidth consumption/latency and alleviating security risks. Furthermore, the distributed computation resources can facilitate the deployment of AI on the edge, and thus capacitate extensive edge intelligence in 6G. Therefore, edge computing is an indispensable supporting technology for 6G.

Despite that the integration of edge computing into 6G is beneficial, new impediments are still to be conquered.

1. Heterogeneity and variability. An increasing number of computational resources are accumulating among the edge nodes due to the constant performance enhancement of mobile devices. High QoS (Quality of Service) and efficient utilization of decentralized resources rely on the adaptive and real-time schedule of heterogeneous resources. However, resource heterogeneity, dynamic network status and strict performance constraints significantly challenge resource scheduling.
2. Mobility and dependability. The 6G network should facilitate high-motion application scenarios, including vehicular networks and aerial networks. In such circumstances, the network nodes are in constant motion. Therefore, it is a daunting job to guarantee URLLC due to the fact that the network topology is time-varying, and the channels are unstable. Novel solutions are yet to be investigated to ensure the reliability of services for mobile network nodes in the dynamic context.
3. Security and confidentiality. In the envisioned 6G network, the edge nodes will accumulate a large amount of data. These data can be not only the foundation for improving the network performance but also an inducement of malicious attacks and privacy violations. Novel security and privacy protection schemes are imperative necessities with regard to 6G edge computing.

#### 2.2.6. Heterogenous Service Request Handling

The future 6G is supposed to accommodate diversified categories of applications, which put forward a high demand for URLLC. Additionally, the reliability and latency of these applications are dramatically influenced by the dynamic environment. As a result, static schemes are unable to adapt to various application scenarios.

The 6G network aims to integrate terrestrial, aerial, satellite and underwater communication networks. Thus, 6G networks inevitably face access requests which are heterogeneous, unstable and unpredictable. Such features of the access requests may significantly affect the service quality of applications that require URLLC. In addition, a myriad of ultra-dense tiny cells could be a common background to 6G communications. In this scenario, it is still an open problem to adaptively manage network traffic without interfering with the locally deployed optimization and/or AI models (for instance, optimization and/or AI models on a base station).

Fortunately, SDN and NFV arise to rescue the situation. SDN decouples the network control logic from the hardware that performs the underlying computation. NFV can establish a certain number of virtual networks based on a single physical network. Moreover, NFV can also integrate virtual or physical devices from different networks into a single virtual network. SDN can collaborate with NFV elegantly by refining the data packet routing process. With the support of SDN and NFV, a set of AI and/or optimization schemes can be deployed into an AP (Access Point)/BS/edge node in an on-demand mode. And thus, the AP/BS/edge node can adaptively and fleetly select the optimal scheme to handle the current service request in real time.

However, it is still a formidable research topic to answer tremendous heterogeneous service requests through the network. In addition, the QoS of the 6G network is a topic requiring holistic considerations, including IRS radio frequency and edge computing [25].

## 3. Investigation of Existing Works

### 3.1. IRS and Spectrum Efficiency

**Figure 3:**
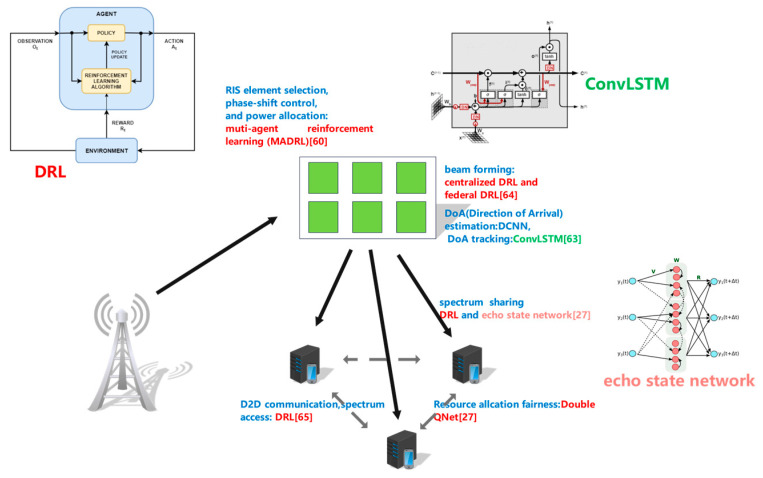
Pursuing high data rate through deep-learning-enabled IRS and spectrum efficiency.

A ubiquitous interactive environment should accommodate enormous simultaneous connections from massive users and support high QoS for every single user. As a result, high bandwidth and data rates are necessities. As shown in Figure 3, representative solutions focus on IRS and spectrum efficiency.

1. Intelligent Reflecting Surface. The forthcoming 6G era is expecting various burgeoning human–computer interactive applications, including not only immersive applications like metaverse but also IoT applications like V2X (Vehicle to Everything) applications. Facing the unparalleled challenges put forward by ultra-high-speed communications and tremendous IoT linkages, THz MIMO-NOMA (massive multiple-input-multiple output non-orthogonal multiple access) has been proven to be an indispensable technology for 6G [60]. The THz MIMO-NOMA system leverages a large-scale antenna array supported by hybrid beamforming infrastructure, which can significantly alleviate attenuation on the THz bands and decrease the hardware complexity and energy consumption. Moreover, users can be categorized into clusters pursuant to the spatial correlation and every cluster is accommodated by a single RF (radio frequency) chain. This clustered mechanism can markedly boost spectral efficiency and connective density. User categorizing can be achieved through clustering algorithms [61,62]. Nevertheless, due to high sensitivity to obscuration, the THz MIMO-NOMA network may be degraded by instability and intermittence caused by either building blockage or life-body blockage. Such instability and intermittence can affect the user experience of reliability-demanding 6G immersive applications. Fortunately, the IRS is a prospective solution to conquer the problems. IRS can dynamically achieve beamforming and thus circumvent blockage by building virtual LoS (Line of Sight) connectivity between transmitters and receivers. Moreover, an intelligent radio context can be set up to observably improve spectrum and power efficiency, as well as induce adaptive scheduling. Currently, IRS has been successfully applied in low-frequency MIMO-NOMA networks. Unfortunately, it is infeasible to directly transplant existing solutions to 6G THz MIMO-NOMA networks. First, existing IRS schemes are unable to handle extraordinarily heterogenous quality-of-service (QoS) demands of 6G users, due to the fact that 6G networks must capacitate diversified devices and services. Second, THz MIMO-NOMA communications are facing a significantly higher probability of unreliability than low-frequency MIMO communications. Third, THz MIMO-NOMA networks conventionally contain high-dimensional channel information. Consequently, the existing centralized and iteratively optimized schemes will result in extremely high complexities and data exchange costs under the unprecedentedly complex THz MIMO-NOMA scenario.

In view of the aforementioned challenges, Xu et al. propose an intelligent reconfigurable MIMO-NOMA THz framework, which takes into consideration two types of heterogenous users: IoT users and SE (super-fast-experience) users. First, the user cluster design: The SE users may face intra-cluster interference. Such interference is completely avoided by adaptively aligning users’ equivalent spatial channels and customizing NOMA decoding sequences pursuant to the QoS demands. Second, beamforming: Highly directional hybrid beams are adjusted by cooperation among APs and IRSs. This adjustment not only guarantees tailored spatial data channels but also ensures active hybrid beamforming and passive beamforming. As a result, inter-cluster and inter-AP interference are alleviated. Third, IRS element selection: The framework adopts a dynamic IRS element selection structure to facilitate hybrid beam reconfiguration. Thus, negative reflections can be circumvented through a power-efficient element-wise ON/OFF control. In this way, the non-ideal discrete phase-shift problem is conquered.

Based on this framework, Xu et al. formulate three key issues into an NP-hard MINLP (mix-integer nonlinear programming) problem: IRS element selection, phase-shift control and power allocation. This problem is non-convex, strongly coupled and extremely complex. The authors convert it into a Dec-POMDP (decentralized partially observable Markov decision process). Eventually, the decision process is resolved using MADRL (muti-agent reinforcement learning).

The IRS is supposed to provide an indispensable hardware foundation for inter active 6G applications. Moreover, the IRSs typically work with THz or mmWave radios. THz radio waves are expected to provide a 100+ Gbps data rate for 6G wireless networks. Under the support of hybrid DAoSA (dynamic array-of-subarrays) beamforming, UM-MIMO (ultra-massive multiple-input-multiple-output) systems can circumvent the distance limitation with lower hardware complexity. Nevertheless, THz DAoSA systems are facing two barriers: millidegree-level three-dimensional DoA (direction-of-arrival) estimation and millisecond-level beam tracking with low pilot cost. 

DoA is an indispensable solution to ensure accurate beam alignment. DoA estimation is conventionally implemented by beamforming training, i.e., the receiver processes a series of pilot symbols with corresponding beams. In addition to the initial beam alignment, DoA tracking guarantees intermittency and reliability under variant channel contexts.

To conquer the barriers, Chen et al. propose an off-grid subspace-based DAoSA-MUSIC (dynamic array-of-subarrays, multiple signal classification), as well as a deep convolutional neural network method for DoA estimation. Moreover, DoA tracking is achieved by analyzing temporal correlations of dynamic channel features. Concretely, the channel variant is explored by an augmented DAoSA-MUSIC-T (dynamic array-of-subarrays, multiple signal classification and terahertz) and a convolutional long short-term memory [63].

As is discussed above, mmWaves are increasingly used in future wireless networks like 5G and 6G, due to the fact that mmWaves can provide abundant bandwidth. Since 5G/6G networks are supposed to facilitate novel services like wearable-device-carried virtual/augmented reality and V2X communications, speedy and efficient beamforming is indispensable in the environment of co-channel interferences and signal attenuation. However, conventional digital beamforming is power-consuming and raises high demand for hardware. In contrast, hybrid beamforming provides comparable performance yet achieves lower overhead and complexity. Nevertheless, most of the existing hybrid methods demand real-time CSI (channel state information), which is captured by either sparse channel estimations or exhaustive/hierarchical searching. Unfortunately, such an estimation or searching scheme may lead to uncertain CSI or require excessive signaling if channel status and cell associations are under dramatic and fast variations. Consequently, they are inefficient in a dynamic and mobile environment. Fozi et al. propose an efficient mmWave beamforming scheme for fast-moving user equipment in the context of channel variations and inter-channel interferences [64]. This scheme has both centralized and distributed processing/training modes and conquers the predicament of frequent handovers of fast-moving user equipment when they are on borderlines among cells and/or not in the line of sight. The centralized mode adopts deep reinforcement learning to alternatively execute training and online beamforming. Nevertheless, this mode faces the disadvantages of heavy communication overhead and phase synchronization among access points. In contrast, the distributed mode leverages federated reinforcement deep learning to locally process data on user equipment and update the deep learning model. Consequently, the communication overhead significantly drops, and phase synchronization is avoided. Experiments show that the scheme dramatically benefits V2I (vehicle to infrastructure) and T2I (high-speed train to infrastructure) communications.

2. Spectrum efficiency. IRSs and enabling techniques like beamforming and DoA estimation build the physical foundations of broad bandwidth and high data rates. In order to fully utilize this underlying support, spectrum efficiency is another vital issue.

Many potential applications like 6G cellular communications are featured with highly dynamic context and limited training data. Consequently, it is undoubtedly vital to design low-training-overhead DRL schemes capable of recognizing the temporal correlation contained in the dynamic context. DRL has been proven to be a promising solution in diversified fields. Fused with RNN (recurrent neural network), DRL can take into consideration the temporal information and thus work in the non-Markovian environment. Whereas, it is a non-trivial job to elegantly train an RNN or DRL model due to the fact that convergence requires tremendous training epochs and iterative tuning of various hyperparameters. Chang et al. leverage DEQN (deep echo stat QNet) to train the DSS (dynamic spectrum sharing) model within a short time interval using limited training data [27]. Experiments show that the proposed scheme can significantly raise the utilization rate of the spectrum.

Inter-device communication is also a prospective technology to facilitate spectral-efficient IoT in 5G/6G networks. Huang et al. refer to inter-device communication as D2D (device-to-device) communication and adopt DRL to improve the spectrum efficiency of D2D-based cellular networks [65]. Moreover, this method can be applied to both uplink and downlink cases. In particular, they elaborate on a time-splitting cellular network in which the D2D nodes and CUEs (cellular users) share the spectrum resources in a time-slotted mode. In addition, D2D nodes can reuse the CUE-preoccupied time slots through an LSA (location-based spectrum access) strategy if the network QoS requirements are satisfied. The main challenge to this method is that D2D nodes have no prior knowledge of LSA strategies or CUE behavior patterns. This challenge is addressed with the support of DRL. Furthermore, resource allocation fairness is explored using the DDQN (double deep QNet) and an extended objective function.

### 3.2. Ultra-Low Latency and Reliable Communication

**Figure 4:**
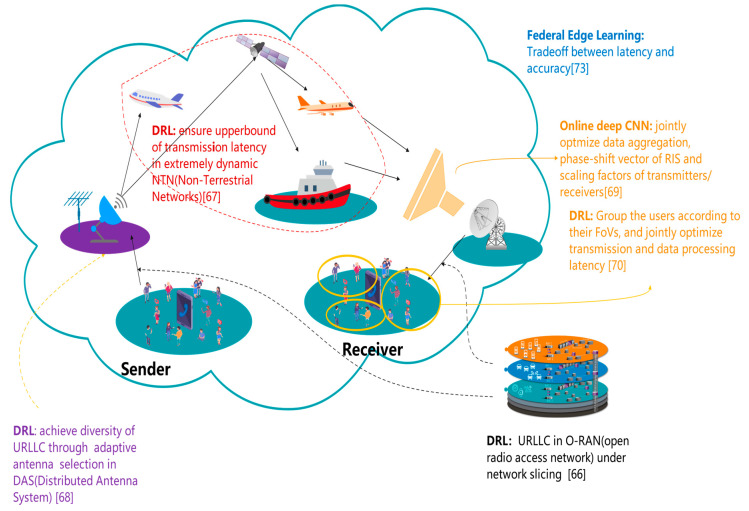
Ensuring URLLC from the following perspectives: radio access, diversity, data processing, coverage and pervasive intelligence.

Low latency is a vital performance metric in human–computer interaction, which significantly influences the user experience. In addition, in some critical scenarios, like distant surgery and healthcare robotics, not only latency but also reliability is neglectable to guarantee QoS or even safety. There have been research works on deep-learning-based URLLC (ultrareliable and low-latency communication) of 6G [24]. As shown in Figure 4, URLLC can be explored from the following perspectives: radio access, diversity, data processing, coverage and pervasive intelligence.

1. Concerns on URLLC. Next-generation communication technologies like 6G highlight the scenario of URLLC. URLLC is indispensable for the development of diversified prospective applications including non-terrestrial communication networks, virtual reality, augmented reality, extended reality, automatic driving, all-region emergency communication, tactile Internet and industrial automatic control. In the traditional communication network, random latency in upper network layers contributes a large fraction to the end-to-end delay. Typical causes of such random latency include queuing, data processing and access delay, while the transmission latency only occupies a minor percentage of the end-to-end delay.

As a result, it brings unparalleled challenges to dramatically lower the upper bound of the end-to-end delay and, meanwhile, maintain high reliability. Recently, enormous potential solutions have been proposed. The following are three representative design concerns. First, URLLC typically requires cross-layer design. Nevertheless, such a design scheme conventionally results in a mathematical model that relies on nonconvex optimization, which induces high time overhead. Such a model can be approximately resolved using deep learning to seek the balance between offline training overhead and online referencing accuracy. Second, integrating the domain knowledge can significantly raise the efficiency of URLLC-oriented deep learning. Third, due to the dynamic nature of the 6G network, the traditional offline-online mode may not necessarily satisfy the performance demands. Ongoing research works are investigating self-adaptive solutions that can automatically explore novel optimization strategies and transfer knowledge to the URLLC applications [24].

2. Radio access and diversity. Filali et al. propose a scheme to achieve URLLC in O-RAN (open radio access network), where the latter is a promising mechanism of computational and communication resource sharing in the network slicing [66]. The network slicing problem is modeled as a single-agent Markov decision process. Subsequently, the process is resolved using deep reinforcement learning. Aiming at URLLC services, both computation slicing and communication slicing should be efficiently accomplished, even if the network contains enormous devices. In order to conquer this predicament, DRL is an efficient solution due to the fact that DRL is capable of handling the curse of dimension.

The work of [66] elaborates on the URLLC of O-RAN, while O-RAN is an enabling technology of network slicing. Network slicing is a prospective solution to heterogeneous QoS requirements. Since 6G is supposed to accommodate various types of interactive applications, the 6G network necessarily meets various QoS requirements, including latency and reliability. Therefore, diversity is a key point to guarantee URLLC. DAS (Distributed Antenna System) is a prospective technology to achieve diversity. Whereas, antenna selection is an unneglectable barrier to efficient DAS. The following two factors dramatically hinder satisfying QoS requirements. First, it is an unavoidable task to select the optimal combination of antennas and UEs out of tremendous base station antennas and UEs. However, traditional solutions like greedy algorithms are merely sub-optimal and yet time-consuming. Second, UEs still need scheduling to optimize the satisfaction rate of QoS requirements.

In view of the challenges, Onishi et al. design an adaptive antenna selection method to simultaneously optimize the combinations of both antennas and UEs [67]. They first model the antenna selection as a Markov chain and then filter the UEs to decrease the amounts of inputs and actions. Subsequently, the optimal combination is identified through DRL.

3. Data processing. Works of [66,68] explore URLLC from the perspective of radio waves and antennas. Based on these works, data processing latency can be further investigated, which is liable to be neglected by conventional research works. Works of [69,70] investigated over the Air Computing [71,72] and data processing latency optimization, respectively.

6G is supposed to enjoy native artificial intelligence, which is featured by services like decentralized FL. And, this prospect requires disruptive innovation of not only the communication and computation paradigm but also the wireless context. AirComp (over the Air Computing) can leverage the waveform superposition properties within a multi-access channel and proposes a prospective solution to ultra-high-speed wireless data aggregation. Bouzinis et al. incorporate distributed AirComp, IRSs and machine learning, so as to reshape the traditional wireless context into an intelligent AirComp context. And, the intelligent AirComp context can execute computational tasks in a thoroughly distributed manner on the physical layer. Moreover, they minimize the error of AirComp by jointly optimizing the phase-shift vector of IRS and scaling factors of transmitters/receivers. Nevertheless, variable coupling and unit modulus constraint result in the non-convex nature of the objective function. Conventional solutions to such non-convex optimization are typically suboptimal. Moreover, such suboptimal solutions conventionally face high time complexity and difficulty of convergence. As a result, the authors propose an online DNN (Deep Neural Networks) to resolve the optimization.

6G is inherently supposed to accommodate various types of applications, and even a single category of application may put forward diverse QoS requirements. For instance, enormous VR devices may work under diversified interference levels, which places barriers to interference management. RSMA (Rate Splitting Multiple Access) is a promising technique for interference management of 6G. RSMA enjoys two advantages: low scheduling complexity and the absence of weak interference assumption. However, in the envisioned 6G VR applications, not only data transmission but also data processing can contribute to latency. Traditional RSMA only concentrates on the maximization of data rate but explores neither the underlying correlation between communication and computation, nor the interplay between the transmitter and end users. Hieu et al. define a joint optimization problem that takes into account the latency, interference management and computational resource management [70]. Then, they transform this problem into a policy optimization problem in terms of DRL. In addition, the PPO (Proximal Policy Optimization) algorithm is adopted to ensure stable training convergence within a huge continuous action space. DNN is used as a nonlinear function approximator to handle heterogeneous input data.

The aforementioned research on URLLC typically focuses on specific technical details. Works of [67,73] take broader views. They explore URLLC from perspectives of larger geographical coverage and more pervasive context, respectively.

4. Geographical coverage and pervasive context. In some time-critical applications like Holographic data transmission, merely “low” latency is not sufficient. Instead, the latency should be “deterministic” and thus ensure reliability. Yu et al. elaborate on the upcoming DetNet-enabled ITNTN (Deterministic Networking enabled Integrated Terrestrial and Non-Terrestrial) and discuss the interaction among bandwidth, latency and computational power [67]. Under the support of NTN (Non-Terrestrial Networks), traffic flows generated by terrestrial network nodes can travel along routines that may comprise UAVs (Unmanned Aerial Vehicles), satellites and aerial platforms like aerostat. The NTN nodes may form uncongested paths compared to the terrestrial network nodes. However, it is a non-trivial job to ensure deterministic performances even if in terrestrial networks. It is even more challenging to obtain determinacy in NTNs. Fortunately, DNSR (Deterministic Network Selection and Routing) is supposed to be a memoryless process, and thus can be modeled as status transitions along Markov chains within a state space. Consequently, they propose a DRL-based scheme for DNSR. The end-to-end latency of integrated holographic flows can be confined to a deterministic range. The aerial platforms are also known as HAPS (High Altitude Platform Stations) [74].

Despite that, FL is a promising technique to boost the native artificial intelligence of 6G, model uploading from devices to the parameter server may become a bottleneck, and thus result in high latency. Consequently, FEL (Federal Edge Learning) is proposed to conquer this barrier. In FEL, the edge server, which is located close to edge devices, plays the role of model aggregator. However, this scheme is a double-blade sword. On one hand, the time overhead of model uploading can be reduced. On the other hand, a single edge server typically covers significantly less edge devices than a traditional FL parameter server, which means dramatic degradation of training efficiency. Sun et al. propose an asynchronous training algorithm for FEL to seek a balance between latency and efficiency [73]. Moreover, how to increase users’ participation in FL is also a vital issue [75].

### 3.3. Energy Efficiency

**Figure 5:**
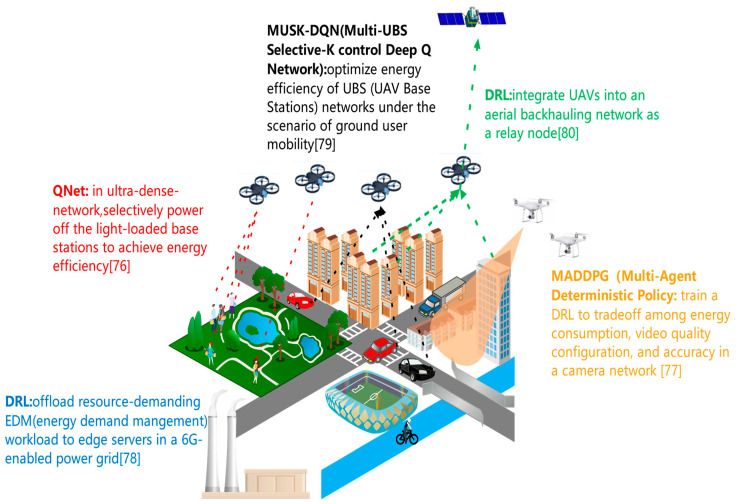
Energy efficiency solutions under the scenarios of smart city and UAV-assisted interaction.

The representative works on energy efficiency are shown in Figure 5.

1. Energy efficiency under the background of a smart city. A smart city is a typical scenario of the envisioned interactive 6G era, where ultra-dense networks are widely deployed. Despite that, UDN (Ultra-Dense Network) has significant advantages, but a boom in power consumption seriously hinders the practical deployment of UDNs. High power consumption induced by the dense deployment of tiny cells has become a key barrier to the target of UDNs, namely achieving a throughput growth of two orders of magnitude in 5G/6G networks. Recently, the sleep mode technique has been proposed in academia. This technique decreases the power consumption of BS by selectively powering off the light-loaded BSs. Nevertheless, it is a challenging task to dynamically determine and convert the modes (working/sleeping) of BSs. The reason lies in the fact that decision-making is extremely time-consuming and frequent mode alterations of enormous tiny cells result in nonnegligible time and power costs. Ju et al. adopt deep QNet to decrease the power consumption of UDNs [76]. They propose a decision selection network to filter inappropriate mode alterations from the action space of QNet model: a feasibility test is used to eliminate the mode alteration that disobeys the rate constraints; the energy test is used to avoid the mode switching that induces excessive power consumption. Experiments show that this scheme can achieve significantly lower power consumption under the rate constraints.

In addition to UDNs, a digital twin is another supporting technology of a smart city. The envisioned sensor network is a cornerstone for building a digital twin of the physical world. It is a daunting job to deal with real-time video from mobile edge devices due to the following reasons. First, the battery capacity and computational resources of edge devices are limited. Second, video frames captured by the edge device are constantly varying. As a result, it is difficult to predict the startup time of a specific task. The work of [77] demonstrates the associations among energy consumption, video quality configuration and accuracy. The authors derive an optimization problem that covers energy efficiency, video quality configuration and offloading decision-making. This optimization problem is intractable due to the complexity of mobile-edge-device video analytics. The state space is yet excessively huge even if the problem is converted into a Markov setting. Therefore, they adopt MADDPG (Multi-Agent Deterministic Policy Gradient) to train a DRL model and thus achieve a superior tradeoff between energy efficiency and other performance metrics, compared to existing works.

Works of [76,77] propose deep-learning solutions to raise the energy efficiency of specific interactive applications under the background of a smart city. Furthermore, deep learning can optimize the power grid which supplies energy to the smart city. SG (Smart grid) system can endow the power grid with higher elasticity and efficiency, and thus fully utilize the bidirectional flows of power and data between the power source and consumer. Aiming at EDM (Energy Demand Management), the SG system is required to process massive data produced by digital sensors and AMI (Advanced Metering Infrastructure): in a power grid, an AMI can adaptively adjust the power provisioning to the shifting demands. The work of [78] proposes a DRL-based scheme to offload resource-demanding EDM workload to edge servers in a 6G-enabled power grid. The computational abilities of smart meters are leveraged to enable autonomous resource provisioning.

2. UAV-assisted communication. UAV-assisted stations or relay nodes can conquer terrain limitations, and thus achieve high flexibility for human–computer interaction. However, it is challenging to integrate UAVs into the communication network due to their limited battery capacity.

UAV-assisted cellular networks are arising as a building block of the envisioned 6G mobile communication. Nevertheless, the battery life of UAV-based devices typically cannot satisfy the requirements of wireless communication. Consequently, frequent switch among UBSs (UAV Base Stations) is unavoidable and hinders seamless and dependable connection. Lee et al. present MUSK-DQN (Multi-UBS Selective-K control Deep Q Network) to optimize the energy efficiency of UBS networks under the scenario of ground user mobility [79]. In MUSK-DQN, the control algorithm is deployed and executed in a ground control station. This method can select *k* UBSs to control in an application-aware way, and thus circumvent the unnecessary energy consumption induced by round-robin control. Moreover, this method can choose an action by considering not only dynamic features caused by ground user mobility but also the status information of all UBSs. 

6G networks are supposed to enable high-speed and low-latency communications everywhere in the world, including the abyssal region. Despite that, satellites can support 6G communications and the distant transmission delay between the satellite, and the end device causes constraints on service capacity. Furthermore, significant growth in sailing-time connections put forward higher requirements on throughput and latency. Hassan et al. orchestrate UAVs into an aerial backhauling network, which plays the role of relay nodes in the marine communication [80]. This network is aided by satellites and littoral base stations. Their proposed scheme formulates the power allocation problem of the 6G-enabled multi-satellite network into a non-convex optimization problem. They propose a DRL algorithm to resolve this problem and outperform not only the water-filling method but also Q-learning.

### 3.4. Security

**Figure 6:**
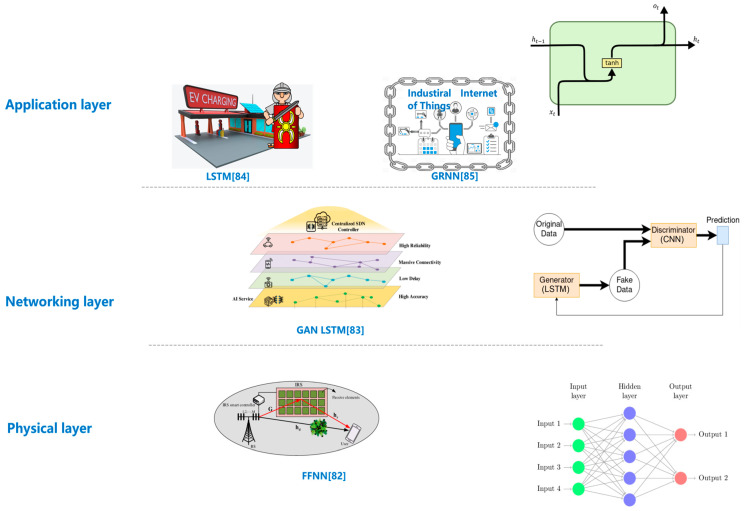
Security solutions on three layers.

The representative works on security can be categorized into three layers (Figure 6).

1. Physical layer. Despite that, interactive 6G applications exhibit significant diversity, security of applications essentially relies on hardware security. Due to the dramatic progresses of programmable meta-material technologies, spectral and power efficiency is increasingly important. It has been an active research topic of 5G/6G networks to integrate IRS into SWIPT (simultaneous wireless information and power transfer) systems [81]. Moreover, an IRS-based SWIPT system is facing security vulnerabilities that can be readily leveraged by eavesdroppers. Thien et al.’s research on the physical-layer security and transmission optimization problems of an IRS-based SWIPT system [82]. And, they presume that a PS (power-splitting) scheme is deployed in the UE. The aim of their research is to maximize the system secrecy rate through jointly optimizing the following variables: transmitter power, PS factor of UE and IRS phase shifts matrix. And, the optimization is accomplished under the constraints of minimum harvested energy and maximum transmitter power. They put forward an AO (alternative optimization)-based scheme to resolve the optimal solutions. The AO-based scheme is an effective solution to either convex or non-convex problems. Nevertheless, this scheme typically induces high time complexity and overhead, due to the fact that enormous mathematic transformations are performed, and a larger number of iterations are required before convergence. As a result, the authors adopt deep learning to train a deep FFN (forward feedback network) model based on labeled data obtained by the AO-based scheme. The deep-learning-based scheme can achieve similar performance to its AO-based counterpart, yet with dramatically low time cost.
2. Networking layer. The heterogeneity of interactive applications necessitates SDN (software-defined networks) and NFV (network function virtualization). Furthermore, SDN and NFV are the foundations of network slicing, which is a promising solution for dealing with heterogeneity. Since network slices must share limited resources, energy efficiency, security and QoS are extremely important performance metrics. As a result, it is vital to address the energy/security/QoS issues under SDN/NFV-aided 6G network slicing environment. However, security is a challenging issue in 6G, due to the fact that traditional methods are not directly portable to 6G networks. Abdulqadder et al. propose a 6G-oriented security scheme based on various state-of-the-art technologies [83]. Firstly, GAN (Generative Adversarial Network) is adopted to achieve deep network slicing. Concretely, GAN can predict the suitable slice and connections for network traffic, pursuant to the capacity, priority and QoS requirements of the slice. Moreover, the DAG (Directed Acyclic Graph) based blockchain technology is integrated to ensure security. The DAG blockchain replaces conventional consensus with the PoS (proof of space) algorithm and conquers the constraints on elasticity and resource overhead in traditional blockchains. Aiming at stronger security, the proposed scheme uses context-aware authentication and secure handover, where the former is based on Markov Decision Making and the latter relies on Weighted Product Model. Afterwards, intrusion packet classification and packet migration are utilized to alleviate the heavy workload on the SDN controllers and switches. The classification and migration are implemented by HyDNT (Hybrid Neural Decision Tree) and HPoHO (Hybrid Political Optimizer with Heap-based Optimizer), respectively. Eventually, the SAC (Soft Actor Critic) algorithm is leveraged to predict the load.
3. Application layer. Despite that, VR and AR can provide an immersive interactive environment, interactions ultimately occur between humans and the real physical world. Moreover, almost any device integrated into the 6G network can interact with users, including traditional industrial devices. However, traditional industrial devices are inherently pregnable to cyberspace attack, when integrated into the 6G network.

The rapid progress of next-generation communication technology dramatically promotes the development of the SCADA (supervisory control and data acquisition) system. However, SCADA is vulnerable due to the fact that it is conventionally a remunerative and tempting attacking target. However, most existing research works only cover the ransomware attack on industrial control systems or SCADA-based systems. Basnet et al. design the deep-learning-powered ransomware detection framework for the SCADA-governed EVCS (electric vehicle charging station) [84]. This elastic and interoperable framework can be deployed to protect various vulnerable points in SCADA-governed EVCS, and each point requires a specific implementation of the framework. In addition, an information-sharing mechanism can ensure that spatially distributed frameworks share the ransomware features once any one of the frameworks recognizes ransomware. Basnet et al. validate the performance of the proposed framework separately using three deep-learning algorithms: DNN, 1D CNN (convolution neural network) and LSTM (long short-term memory). The proposed framework achieves high accuracy with all of the adopted deep learning algorithms.

The IIoT (Industrial Internet of Things) is one of the cornerstones of Industry 4.0. What is more, 6G is a promising candidate to guarantee indispensable features of IIoT, including high reliability and low latency. An increasing number of network devices are being integrated into the IIoT environment, while these devices may locally store raw data and mutually share the data. However, storage and sharing are implemented with the absence of any direct human manipulations. As a result, security concerns inevitably arise, including data integrity and privacy. Hilal et al. point out the disadvantages of conventional IDS (intrusion detection system) in a 6G-enabled IIoT context and propose a private preserving blockchain with a deep-learning model for the IIoT under the 6G environment [85]. This blockchain system leverages BGRNN (bidirectional recurrent neural network) to recognize intrusions, and the hyperparameters are fined tuned with the assistance of CGO (Chaos Game Optimization). In addition, BEIC (blockchain-enabled integrity check) is adopted to guard against misrouting attacks that tamper the OpenFlow rules of SDN-based IIoT, while SDN is a common virtualization framework to support 6G networks. The proposed solution is validated on the ICSCA (Industrial Control System Cyber-attack) dataset. Similarly, the work of [86] proposes a security solution to energy storage systems of IIoT, using deep learning.

### 3.5. Edge Computing

**Figure 7:**
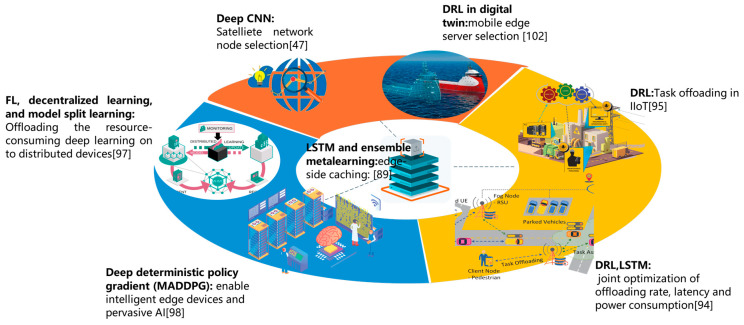
Key technologies of edge computing in interactive 6G context.

Due to the inherent large-scale distributed nature of interactive 6G systems, it is an indispensable issue to fully utilize the computational resources of edge devices other than only relying on the cloud server [87]. This context brings some significant challenges, including but not limited to edge-side caching, node/server selection, task offloading, decentralized learning and pervasive intelligence (Figure 7).

1. Caching. Efficient edge-side caching is a cornerstone of edge computing. To address the urgent challenges to IoT applications, edge-side caching has been proven to be a prospective method to boost application performance in terms of time overhead and power consumption. Whereas, cache capacity is limited in edge-computing scenarios. Moreover, user preferences are frequently changing over both minor and large time scales under real time and interactive contexts, like Twitter and Meta. It is still a challenging task to set up an efficient universal caching framework for diversified user requirements. Nyuyen et al. design a novel content caching scheme to ensure a high-hit ratio through adaptive prediction in variant network and user context [88]. They adopt a hierarchical online learning framework to support a proactive content caching control scheme and thus achieve adaptive caching. This scheme can handle the frequent fluctuations in content popularity and user preference. The online learning framework comprises local LSTM learning, as well as ensemble meta-learning. With the assistance of STL (seasonal-trend decomposition and loess)-based preprocessing, the LSTM is used to extract temporal-specific features and deal with the time fluctuations in popularity. As a result, the dynamic content preferences of each user group are identified in real time. Subsequently, a regression-based meta resembling learning method is adopted to convert the previously resolved multiple demographic user preferences into a unitary online caching scheme. This scheme relies on an online convex optimization framework and achieves sublinear-regret performance.

Based on the cached data, more details can be drawn on the user preferences. Gao et al. propose an NCF (Neural Collaborative Filtering) model to discover user preferences from biased user behavior sequences [89]. They map the data into an embedding space using a self-attention mechanism. Taking the embedding as input, GMF (Generalized Matrix Factorization) and MLP (Multilayer Perception) are adopted to discover user preferences. Experiments on the two real-world datasets MovieLens [90] and Amazon Beauty validate the efficiency. Similarly, Dib et al. convert the Twitter post text into vectors by LDA (Latent Dirichlet Allocation) and input the vectors into LSTM model [91]. The trained model can provide recommendations and outperform state-of-the-art models like average-LSTM and time-LSTM.

2. Node/edge server selection. Node/edge server selection is an important problem in terms of the collaboration among edge nodes and edge servers (like task offloading), etc.

5G and 6G are promising high-speed communication technologies that adopt satellites and CPS (cyber-physical systems) to combine aerial data, terrestrial data and maritime data. Tremendous applications utilize satellite data to handle user requests and produce appropriate responses. As a result, massive satellite data are continuously accumulating. Satellite CPS data imply dramatic value with regard to academic research, industry and commerce. However, the performance of traditional inter-satellite communication is constrained by high latency and low data utilization. SEC (smart edge computing) is a promising solution to fully take advantage of the next generation communication technologies and conquer the constraints. Mohammed et al. construct a logic ring to facilitate satellite CPS with SEC [92]. Within this logic ring, SDN and NFV are used to select edge nodes and cloud nodes in the smart edge computing context. Therefore, the satellite CPS is abstracted as a software system. The inner operations (like node selection) of this system can be formalized as an optimization problem. Afterwards, the problem can be resolved by a deep convolution neural network based on abundant manually labeled training data.

DT (Digital Twin) and mobile-edge computing are two cornerstones of 6G. Nevertheless, collaboration on the edge side is still an open problem. Efficient edge collaboration can significantly boost the system’s performance. Liu et al. investigate offloading tasks to collaborative MES (mobile-edge servers) in a mobile communication context, with the aid of DT [93]. They propose a DT-assisted strategy to select MES and intelligently offload tasks. Concretely, MES selection is accomplished based on channel state information and blockchain. MES with authenticated data consistency and high-reliability links tend to be selected. Subsequently, they model the task offloading process as a Markov decision process and map the physical devices into virtualized context. As a result, the intelligent offloading model is trained using a neural network with the absence of power and resource consumption in physical devices. Finally, they formalize and mathematically optimize the system to minimize power consumption and delay.

3. Task offloading. Edge computing has recently witnessed dramatic progress in both academia and industry. In addition, edge computing is believed to be a prospective scheme to improve the information processing ability of edge devices for the 5G/6G networks. Owing to the wide application of enormous low-power dissipation intelligent devices and the rapid increase of data volume, it is a vital job to offload intensive computation to edge devices. Aiming at jointly leveraging the advantages of both deep RNN and LSTM, Kashyap et al. propose the DECENT framework (Deep-Learning-Enabled green Computation for Edge centric Next generation 6G Networks) [94]. They model data offloading as a Markov decision process. Since the 6G network accounts for countless states and actions, it is a daunting job to record entire Q-values in a table. They adopt LSTM to simplify the original state-action space and produce an approximate state-action space. Moreover, this process jointly optimizes the following performances: power consumption, computation overhead and offloading rate for network utility in the 6G context. This proposed algorithm boosts the training stage and achieves a higher convergence speed.

The 6G technology is an indispensable cornerstone to achieve the transformation from “interconnected things” to “interconnected intelligence”. Consequently, 6G networks will undoubtedly undergo record-breaking reformation. As a vital potential application scenario of 6G, IIoT contains various network nodes including controllers, sensors and actuators. In addition, information distributed in the industrial field can be gathered and become the source of industrial intelligence. Gong et al. boost the task scheduling and resource allocation of IIoT using MEC (multi-access edge computing), which is a prospective technique of 6G networks [95]. They use reinforcement learning to jointly optimize task offloading and resource allocation, where the objective function reflects the weighted sum of latency and power consumption. Moreover, they adopt a convex programming method named isotone action generation to quantize the task offloading actions into binary values. In order to stabilize this quantizing method and alleviate over-fitting, the DRL agent can be retrained through experience replay and stochastic sampling. In addition, a higher value of the action aggregation number implies a larger decision space and thus induces a heavier computational workload. The work of [95] adopts the concept of rolling horizon control and periodically updates the number of action aggregations throughout stepwise reinforcement learning. In this manner, the rise of action aggregation numbers slows down without significantly affecting the learning performance.

4. Decentralized learning and pervasive intelligence. The rapid development of artificial intelligence has dramatically boosted the evolution of wireless networks. As a promising technology, 6G will undoubtedly induce a revolution of wireless networks from “connected devices” to “connected intelligence”. AI/data-driven technologies are essential to almost every 6G applications [96]. Nevertheless, mainstream deep-learning techniques typically demand massive computational resources and high communication bandwidth and thus induce non-negligible time overhead, power consumption and privacy risks. By integrating machine learning abilities into the network edge, AI-enabled edge devices rise up as a ground-breaking solution to fuse diversified cutting-edge technologies including digital sensors, next-generation communication, computation and AI. Letaief et al. show a vision for adaptive and reliable edge AI systems, with integrated consideration of wireless communication solutions and distributed machine learning models (FL, decentralized learning and model split learning) [97]. They propose new design standards for wireless networks, service-driven resource allocation strategies and a supportive framework for edge AI. Moreover, they also discuss standardization, platforms and application context, facilitating industrialization and commercialization.

The next-generation mobile network may face a significant reformation in a vertical industrial context. In this context, the DENs (deep edge networks) has emerged as a key technology for the smart resource management of 3C (computing, caching and communication). DEN can endow the edge nodes with computational and decision-making abilities. Moreover, DEN can incorporate computation resources with wireless communication through instantaneous collaboration to produce the vision of pervasive intelligence. Gong et al. propose an intelligence resource management framework for DEN, which can offload task scheduling onto edge nodes and thus give rise to instantaneous integration of computation and communication [98]. This framework covers two ubiquitous scenarios: single-edge scenes and multiple-edge scenes, where they integrate wireless communication and computation based on real-time intelligent collaboration. In the single-edge scene, the optimization framework is designed using deep reinforcement learning. This framework can collaboratively optimize the task dispatching, power consumption and processor cycle frequency in a dynamic channel environment. Consequently, the convergence performance is improved, and the latency is decreased. In the multiple-edge scene, they adopt a MADDPG algorithm to minimize overall power consumption and latency. In this algorithm, the training phase is in a centralized mode, while the inference is deployed in a distributed mode. In this manner, the interference in multiple-edge scenarios can be relieved. In addition, the algorithm achieves quasi-optimal overhead for multiple agents under a metabolic and mutual interference network context.

### 3.6. Heterogenous Service Requests

**Figure 8:**
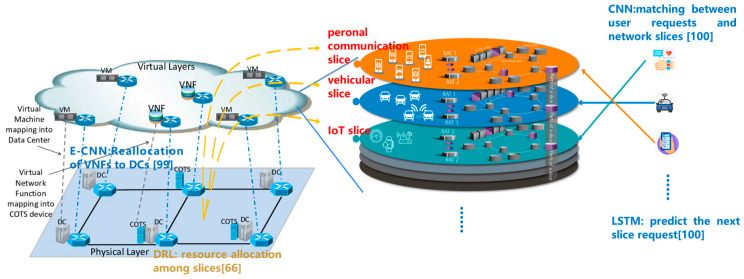
Solutions to heterogeneous QoS requirements of interactive 6G applications.

The diversity of interactive 6G applications proposes heterogeneous requirements on SDN/VNF and network slicing (Figure 8).

1. SDN and VNF. IoT vertical applications supply out-of-the-box utilities to address the challenges raised by diversified domains. Such vertical applications enjoy broad prospects yet put forward requirements in latency awareness, privacy protection and scalable intelligence. With the rapidly increasing amount of IoT links, intelligent and real-time-deployable VNF configurations will set the cornerstone for the persuasive network context. Emu et al. comprehensively take into consideration the prospective service configuration requirements [99]. They emphasize the urgency of surpassing the conventional service deployment architecture and propose to allocate VNFs using edge cloudlet mini-scale data center. Emu et al. systematically integrate various technologies to achieve automatic VNF configuration and apply deep learning to enhance the VNF configuration model. This model opens a new way to handle 6G-network challenges through deep learning.

VNF is expected to deal with some extremely challenging issues in the 6G IoT. For example, the balance between reconfiguration overhead and performance benefits. The VNF means to map physical network devices to virtualized ones, and thus gain high flexibility of network reconfiguration. Reconfiguration is an indispensable ability to adaptively adjust to the dynamic environment in the 6G IoT. Reconfiguration can induce performance benefits yet still causes unavoidable overhead. As a result, a reasonable trade-off is required between reconfiguration overhead and performance benefits.

Their model contains two domains: cloudlet and RAN (radio access network). The former comprises cloudlets, i.e., mini-scale data centers that accommodate VNFs; the latter contains eNBs (viz. base stations) that interact with end users. Every cloudlet can host a certain number of eNBs, and an eNB can logically consist of a certain number of VNFs. VNFs affiliated to the same eNB may be distributed among different cloudlets. In the work of [99], VNF reconfiguration refers to reallocating VNFs to cloudlets to achieve improved performance (for example, lower latency) while taking into consideration the tradeoff between performance benefits and reconfiguration overhead. Due to the continual mobility of users and the dynamic network environment, such reconfiguration can be frequently executed.

Allocating VNFs to DCs (Data Centers) can be reduced to an optimum problem. IPL (Integer linear programming) is a common solution to such problems. However, ILP is typically extremely time-consuming and thus not suitable for real-time online reconfiguration. As a result, the work of [99] fabricates a training dataset through ILP and trains the E-CNN (ensemble CNN) model. Consequently, the decision of real-time online reallocating is made through referencing by the trained E-CNN model.

2. Network slicing. As is aforementioned, the transition from the 4G/5G to 6G is evolving from “connected devices” to “connected intelligence”. As a result, 6G networks inevitably face enormous application requirements, which may be significantly different. For example, a smart sensor network typically puts forwards high demand on network capacity while being tolerant to low bandwidth and high latency. By contrast, autopilot systems require an instantaneous response. In other words, 6G networks must simultaneously hold various applications that may raise different or even contradictory QoS (quality of service) requirements. Network slicing enables 6G to answer the challenge by establishing multiple virtual networks on a single hardware infrastructure. We can ensure QoS by reconfiguration and optimization of the networks. Machine-learning-enabled 6G networks can achieve intelligent decision-making and efficiently handle slice failures. Khan et al. propose a slicing model using CNN and LSTM. CNN supports accurate slice assignment to both known and unknown devices and copes with slice failure. LSTM is used to predict slice requests, the workload of the network and the probable slice failure [100]. Simulation on NS2 validates the performance of the proposed model.

Every slice can be viewed as a real network within its lifespan. As a result, common issues of a network are still to be discussed for network slicing. The work of [66] proposes a solution to guarantee low latency and reliability under network slicing. In addition, the work of [101] investigates on fairness of package scheduling. The 6G wireless network is facing tremendous technical challenges, including various design tradeoffs and performance optimization. Artificial intelligent methods like deep learning can train a model in a large parameter space based on enormous training data labeled by human experts. As a result, tradeoffs and optimization (or quasi-optimization) can be achieved through inference on the model. In the artificial intelligent model, a certain setting of model parameters represents the inference path from the model input to the output, and such a parameter set indicates a solution to a specific tradeoff or optimization problem. Due to the large volume of the parameter space, the model is capable of providing diversified solutions to meet the various tradeoff or optimization requirements. Consequently, 6G wireless networks tend to be inherently AI-enabled. Bhandari et al. explore the ethical aspect of 6G wireless network design and propose a scheme for package scheduling fairness [101]. This scheme is dedicated to avoiding an unfair situation where network nodes with better links tend to be selected to transmit data, while nodes with worse links may not be selected. The scheme leverages deep CNN to categorize the network dataset based on link quality between the connecting BTS (base transceiver station) and UE. In addition, data combination methods are designed to integrate the categorized dataset such that nodes with poor link quality are still included in the data transmission routine.

## 4. Problem Study

### 4.1. Systematic Concerns

We divide the 6G communication architecture into three layers: hardware layer (physical layer), carrier-wave layer (networking layer) and application layer. The hardware is the physical generator and receiver of carrier waves. The carrier wave carries the information and is tightly related to the channel (or in other words, the propagation environment). The AI-enabled applications are built upon the former layers. The former two layers are the physical foundation of 6G communication and play a fundamental role in broad bandwidth, high data rate, URLLC and energy efficiency. Other performance metrics (like security, edge computing and heterogeneous QoS) are closer to applications.

Due to the fact that 6G is still in its infancy, most representative works mainly confine their research within a specific layer. However, we are expecting more cross-layer works, with the rapid development of 6G. Take security as an example, the application layer may adopt deep learning to recognize malicious user behaviors; the carrier-wave layer can apply deep-learning-based encryption coding; the hardware layer could integrate deep-learning-enabled security chips. Moreover, a fusion of the solutions on each layer may bring a higher effectiveness/cost ratio. 

In terms of the existing representative works, we can find out that DRL or similar methods account for a high proportion in terms of each layer. Table 1 categorizes the representative works in terms of the adopted deep-learning methods. From the table, we can see that DRL or other Markovian-decision-based deep learning methods are broadly applied and cover almost all challenges to 6G. That is due to the advantages of Markovian-decision-based methods, including but not limited to the following. First, a holistic view. Such methods are dedicated to maximizing the long-term rewards other than concentrating on specific local subtasks. In other words, this kind of method is skillful at trading short-term rewards for long-term rewards. Second, the absence of a separate data collection phase. In Markovian-decision-based methods, training data are naturally gathered through the action–reward interactions between the agent(s) and the environment. There is no independent data collection phase in the algorithm. Thus, the workload of the algorithm can be significantly relieved. Third, inherent adaptiveness. Markovian-decision-based methods are inherently designed to respond to the constantly time-varying environment. The time-varying nature of such a method enables adaptiveness.

The aforementioned characteristics exactly satisfy the requirements of human–computer interactive 6G applications. First of all, 6G aims to construct a human-centric pervasive intelligent world and thus tries to integrate almost all digital devices into a whole and pursue holistic optimization of performances. Such optimization of a complex system requires tradeoffs among subsystems, which falls in the adept domain of DRL and similar methods. Second, 6G is yet a series of novel technologies on the vision. The development of backbone technologies like IRS is still in progress and not yet broadly applied in industry or business. As a result, it is difficult to obtain abundant training data under many application scenarios. While the naturally obtained training data by Markovian-decision-based methods just alleviate this dilemma. Third, the characteristics like broad bandwidth and URLLC put forwards strict demands on the radio wave propagation environment of 6G, which is typically time varying. The adaptiveness of Markovian-decision-based methods shows dramatic advantages in simulating and addressing such problems.

### 4.2. Source of Training Data

Despite the advantages of Markovian decision-based methods, such methods are only applicable to the Markovian environment. In the non-Markovian environment, the memoryless status transition paradigm no longer works. In addition, even in the Markovian environment, a high-dimensional large action space may significantly slow down the learning process toward the final acceptable routine.

Consequently, some applications adopt the conventional training-reference deep-learning methods. In these scenarios, the source of training data is a vital topic. Typical sources of training data include but are not limited to simulation, traditional analytical method solutions and existing datasets.

First, under some application scenarios, training data can be generated through simulation. Due to the infinity of the solution space, Monte Carlo simulation is adopted to generate training data for the direction-of-arrival estimation [63]. Analogously, numerical-simulation-generated data are used to train a CNN, which resolves a joint optimization problem of over-the-air-computing [69]. Similarly, a CNN-based data package scheduler can be trained by a numerical-simulation-generated data [101]. The work of [83] produces training data for GAN using the TeraSim of the NS3 simulator.

Second, 6G applications can borrow existing datasets in certain contexts. Existing ransomware datasets (from the Virus Total website) [103] can be used to train CNN and LSTM for malicious software detection [84]. Deep learning models trained by existing datasets exhibit acceptable performance in experimental validation. In addition, the DeepSlice dataset [90] is used to train CNN and LSLTM, aiming at the slice prediction [100]. Works of [85,88] also adopted ready-made datasets. However, the datasets of these works have been collected or generated in the 4G or 5G era. And thus, they are not 6G-specific.

Additionally, analytical methods can be adapted to generate training data for deep learning under certain scenarios (as discussed in Section 4.3).

### 4.3. Relationship between Analytical and Deep Learning Methods

Deep learning can still play a vital role even if traditional analytical solutions are available. On one hand, some problems are NP-hard in the analytical solution context [60,69]. On the other hand, in some applications, the analytical methods result in excessively high computation complexity even if they can reach the solutions in the polynomial time [82,99]. In both of the two scenarios, deep learning can serve as an alternative that achieves acceptable accuracy with dramatically low computation complexity.

Furthermore, deep learning methods can tradeoff between time complexity and accuracy on some analytical method-solvable problems. The reallocation of VNFs among cloudlets can be formulated as an ILP (Integer Linear Programming) problem. However, traditional analytical solutions to ILP are excessively time-consuming, and thus not directly applicable to highly real-time VNF context [46]. As a result, conventional ILP is used to generate analytical solutions, which serve as labeled training data. Subsequently, an E-CNN is trained as a quasi-optimal solution with a significantly short response time. In the method of [82], conventional analytical AO (alternative optimization) is adopted to generate labeled data to train FFNN.

### 4.4. Frequently Used Techniques

Summing up the representative works from another perspective, we can find some common schemes, as shown in Table 2.

First, multi-objective optimization. Due to the characteristics like sensitivity to signal blockage, the antennas and IRS of THz or mmWave communication exhibit high complexity. As a result, overall performance of the antenna/IRS replies on tradeoff among various parameters. In addition to antennas and IRS, 6G applications put forwards demands on various performances including energy efficiency, security, etc. Deep learning can train enormous network parameters in pursuant to the mapping relationship between samples (status and specific requirements of a 6G application) and labels (settings to satisfy the requirements).

Second, the simplification of DRL decision space. DRL and other Markovian-decision-based methods are broadly applied in current representative 6G-oriented research works, due to dramatic advantages like the absence of a separate data collection phase. Nevertheless, a real-world application typically generates a large decision space, which significantly slows down the training. In order to seek a balance between training error and speed, the decision space can be filtered by a decision selection network [76], simplified by LSTM [94] and constrained to a low growth rate by a rolling horizon control [95].

Third, distributed deep learning. Typically, deep learning algorithms need abundant training data (either from a separate data collection phase or naturally obtained during the training phase) to shape the neural network parameters. The 6G communication system is inherently a distributed one and thus enables distributed data storage and distributed computing. Although distributed training can be achieved through direct data shared among network nodes, this schema induces problems like high bandwidth consumption and privacy invasion. Direct data sharing among network nodes requires careful orchestration [104]. Federal learning can obtain model-by-model aggregation. In this manner, the bandwidth consumption and privacy problems are resolved due to the fact that only local models (other than local data) are shared [64]. In addition to federal learning, multi-agent DRL also well fit into the distributed mode. This kind of DRL aims at multi-objective optimization by adopting more than one agent [60,77,97,98].

In addition, generating training data using analytical methods is still an important technique (as discussed in Section 4.3).

## 5. Open Problems

Deep learning exhibits fantastic prospects in terms of addressing the challenges to 6G systems. However, the fundamental hurdle to physical layer implementation is still to be investigated. Efficient and reliable THz communication puts forward strict demands on enormous physical-layer factors such as the material composition of chips, signal generator, modulation and demultiplexing and so on. Upper-layer designs and applications would be unpractical in the absence of a well-orchestrated physical layer.

1. Material of chip manufacture. Nowadays, it is almost a consensus of both academia and industry that THz communication will be the cornerstone of 6G. However, traditional electronic chips are facing barriers due to their physical limitations. Currently, the THz chips are hindered by some serious disadvantages including but not limited to the following: constrained data rate, crosstalk, scattering loss and inadequate tunability. Phototunable topological photonics open a way to rescue this situation. Future practical 6G communication relies on the fundamental support of semiconductor technologies [105] or even nano technologies [106]. CMOS (complementary metal oxide semiconductor) is a promising type of material for photonic chip manufacture. In the work of [107], an optical governable demultiplexer is achieved through topological protection and a critically coupled high-quality cavity. And thus, two carrier-waves can be modulated into signals with the absence of crosstalk. This demultiplexer is built using a Silicon Valley photonic crystal and can enhance compatible THz communications. Furthermore, integrating photonic components into a single chip is still a challenging task [108].
2. Stable THz Source. The stable THz source is indispensable to precision-demanding THz applications such as tactile communications, millimeter wave radar and onboard communications. Nonetheless, constructing stable and precise THz sources is a challenging task in terms of ensuring low-phase noise and sufficient frequency stability. The work of [109] applied photonic technologies and designed a THz synthesis solution to achieve optical frequency comb stabilization within the THz region.
3. THz signal demultiplexing. Due to the massive-connection vision of 6G communications, frequency bands are becoming scarce resources. Schemes like MIMO (multi-input and multi-output) adopt space-division multiplexing to pursue full utilization of the spectrum. Nevertheless, simultaneous signal transmission using closely spaced frequencies conventionally results in spectral congestion and induces problems like crosstalk. The BSS (blind source separation) can distinguish and recover unknown signals out of their mixture, with minimum prior knowledge. Existing traditional electronic BSS solutions can only work efficiently under narrow-band and low-frequency scenarios. Due to the physical limitation of RF (radio frequency) technology, electronic BSS is impeded to handle broad-bandwidth applications. The work of [110] explored the advantages of optical communication and proposed a mirroring-weight-bank-based BSS chip which achieves high-resolving power.
4. Incurred security issues in the channel. Under the THz communication environment, broad bandwidth and high frequency induce some unneglectable challenges to be investigated. Such challenges include but are not limited to the following. First, high-grain antenna design; second, atmospheric attenuation during high-frequency propagation; third, radio wave scattering and material absorption. Such challenges require novel solutions, yet potential solutions may induce even new challenges. For instance, the Silicon-CMOS-based dense reflect arrays can be a promising scheme to reshape the propagation environment and resolve the issue of the THz wave blockage [111]. Nevertheless, the reflect arrays may be vulnerable to new security threats like meta-surface in the middle attack.
5. Potential deep-learning-enabled directions. Deep learning methods are almost applicable to all realms due to the current empirical paradigm. With regards to the material composition of chips, the novel CMOS material is critical for high-frequency chip manufacture. Nevertheless, the trial-and-error cost is significantly high in traditional material science and engineering. Fortunately, the work of [112] has proven the feasibility of generating and screening semiconductor materials via GAN-based deep learning.

In terms of high-frequency signal demultiplexing, photonics may play an important role. Yun et al. proposed to identify the complicated nonlinear associations between the design space and photonic performances [113]. Ashtiani et al. design a chip-scale photonic deep neural network for image classification [114]. Ma et al. even orchestrate a data-driven design method for the photonic structure [115].

It seems natural to apply deep learning to simulate physical laws like photonic dynamics, due to the empirical and data-driven nature of deep learning. However, the simulation accuracy may be disappointing if we directly migrate deep learning to universal physical laws without any domain-expert modification [116]. Therefore, there is still a long way to go.

## 6. Conclusions

In this paper, we investigated representative deep-learning solutions to the main challenges raised by the envisioned interactive 6G era. We also discussed the open problems. Due to the ultimate target of “connected intelligence” for human-centered interaction, the 6G communication system is featured by physical-layer innovation, heterogenous subsystem integration, device virtualization and adaptiveness to joint performance metrics. These complex requirements place high barriers to traditional analytical methods. Fortunately, deep learning methods can circumvent the problem of interpretability and encapsulate decision-making knowledge into enormous neural network parameters. And thus, deep learning is possibly the most promising solution to generalized 6G problems up to now.

Existing deep-learning solutions have been explored to address the aforementioned challenges and have achieved significant progress. Nevertheless, further problems are still to be addressed. For example, physical-layer issues like chip material and stable THz source require joint efforts of both physics and engineering. Borrowing deep learning in such research topics is extremely challenging due to the necessity of reshaping deep neural networks by general physical laws. Another issue is holistic solutions. Most of the existing work only applies deep learning to a specific problem of 6G. While a holistic solution should consider the overall system from the physical layer throughout the application layer. Such holistic solutions could be a vital step towards practical human-centric interactive 6G systems.

## Figures and Tables

**Table 1:** Representative works are categorized by deep-learning methods.

Deep Learning Method(s)	Challenge(s) Addressed	Focused Performance Metric of 6G	Representative Iterature
DRL (Deep Reinforcement Learning), DDQN (Double Deep QNet)	Device-to-device communication: lacking prior knowledge of cellular users in Location-based Spectrum Access (LSA) (resolved by DRL); fairness of resource allocation in inter-device communication (resolved by DDQN)	High data rate and broad bandwidth	[65]
QNet	In ultra-dense networks, selectively powering off light-loaded base stations to raise power efficiency	Power efficiency	[76]
MUSK-DQN (Multi-UBS Selective-K control Deep Q Network)	Optimizing energy efficiency of UBS networks under the scenario of ground user mobility	Power efficiency	[79]
Centralized DRL	Beam forming of high-speed mobile devices	High data rate and broad bandwidth	[64]
DRL based Federal Learning	High communication overhead in the centralized DRL	High data rate and broad bandwidth	[64]
Muti-agent DRL (MADRL)	A NP-hard MINLP (mix-integer nonlinear programming) problem: IRS element selection, phase-shift control and power allocation	High data rate and broad bandwidth	[60]
DRL trained by MADDPG (Multi-Agent Deterministic Policy Gradient)	Tradeoff among energy consumption, video quality configuration and accuracy in a camera network	Power efficiency	[77]
DRL-based FL (Federal Learning), Decentralized Learning, Model Split Learning	Excessively high time over-head, power consumption and privacy risks in edge computing	Edge computing	[97]
MADDPG (Multi-Agent-assisted Deep Deterministic Policy Gradient)	Integration of computation and communication into edge nodes of the deep edge network, pursuing pervasive intelligence in edge computing	Edge computing	[98]
DRL, RNN, echo state network	DSS (Dynamic Spectrum Sharing) in 6G: highly dynamic but limited training data (resolved by DRL); temporal data handling in a Non-Markov environment (resolved by RNN); high training overhead of RNN (resolved by echo state network)	High data rate and broad bandwidth	[27]
DRL	Modeling of network slicing in O-RAN (open radio access network)	URLLC	[66]
DRL	Ensuring low transmision latency in high dynamic NTN (Non-Terrestrial Networks)	URLLC	[67]
DRL	URLLC of DAS (Distributed Antenna System)	URLLC	[68]
DRL	Integrate UAVs into an aerial backhauling network, which performs as relay nodes in the marine communication	Power efficiency	[80]
DRL	Offload resource-demanding EDM (Energy Demand Management) workload to edge servers in a 6G-enabled power grid	Power efficency	[78]
DRL training in the digital twin of 6G networks	Execessively high resource cost in training DRL in terms of mobile server selection.	Edge computing	[102]
DRL, PPO (Proximal Policy Optimization), DNN (Deep Neural Network)	Joint optimization of 6G VR applications, covering the latency, interference management and computational resource management. Modeling of the optimization problem (resolved by DRL); instability of training convergence within a huge continuous action space (resolved by PPO); heterogenity of input data (resolved by DNN as a nonlinear function approximator)	URLLC	[70]
DRL, LSTM	Joint optimization of task offloading rate, power consumption and computational overhead: offloading scheme selection by DRL; simplification of original state-action space by LSTM.	Edge computing	[94]
DRL	Optimization of task offloading and resource allocation in IIoT (Industrial Internet of Things)	Edge computing	[95]
Deep CNN (Convolution Neural Network) and convLSTM (Convolution Long Short-term Memory)	DoA (Direction-of-arrival) estimation (resolved by DeepCNN), millisecond-level beam tracking (resolved by convLSTM) and training data are obtained by Monte Carlo simulation	High data rate and broad bandwidth	[63]
DNN (Deep neural network), CNN (convolution neural network) and LSTM (long short-term memory)	Ransomware recognition in SCADA-governed electric vehicle charging station (EVCS), training data are obtained from the VirusTotal website	Security	[84]
Online Deep CNN	Minimizing error of over-the-air computing through joint optimization of following factors: phase-shift vector of IRS and scaling factors of transmitters/receivers; training data are obtained by numerical simulation	URLLC	[69]
E-CNN (Ensemble Convolution Neural Network)	Reallocation of VNFs among cloudlets: training data are obtained by traditional ILP (Integer Linear Programming); E-CNN is trained based on the data	QoS heterogeneity	[99]
CNN, LSTM	Slice prediction and assignment: slice requirement prediction by LSTM; slice assignment by CNN; the already-made DeepSlice dataset is used as training data.	QoS heterogeneity	[100]
CNN	Fairness of data package scheduling; training data are generated by numerical simulation.	QoS heterogeneity	[101]
BiGRNN (Bidirectional Gated Recurrent Neural Network), CGO (Chaos Game Optimization)	Industrial Internet of Things intrusion detection: intrusion detection by BiGRNN; hyperparameter tuning by CGO; training is based on already-made dataset.	Security	[85]
GAN (Generative Adversarial Network)	Prediction of the suitable slicing under the SDN/VNF environment, according to performance metrics including security; training data are generated by TeraSim simulator (in NS3)	Security	[83]
FFNN (FeedForward Neural Network)	Maximizing the secrecy rate of SWIPT (simultaneous wireless information and power transfer) systems through joint optmization of the following factors: transmitter power, PS factor of UE and IRS phase shifts matrix. (train a deep FFNN based on labeled data obtained by the traditional analytical AO (alternative optimization-based scheme))	Security	[82]
LSTM, Ensemble Metalearning	Edge side local caching in MEC (Multiple/Multi-access Edge Computing): dynamic content preferences of each user group are identified by LSTM; combination of multiple demographic user preferences into caching scheme by Ensemble Metalearning; training data are from real word datasets MovieLens	Edge computing	[88]

**Table 2:** Frequently used techniques in deep-learning-based 6G solutions.

Typical Technique	Summary	Representative Work
Multi-objective optimization	Phase-shift vector of IRS and scaling factors of transmitters/receivers	[69]
	IRS element selection, phase-shift control and power allocation	[60]
	Transmitter power, PS factor of UE and IRS phase shifts matrix	[82]
	Task offloading rate, power consumption and computational overhead	[94]
	Task offloading and resource allocation	[95]
	Latency, interference management and computational resource management	[70]
Simplification of DRL decision space	Adopt a decision selection network to filter inappropriate mode alterations from the action space of QNet model	[75]
	Adopt LSTM to simplify the original state–action space and produce an approximate state–action space	[94]
	Adopt the concept of rolling horizon control to slow down the growth of action aggregations	[95]
Deep learning as an alternative to traditional analytical methods	Obtain training data by AO (alternative optimization)-based scheme) to train a FFNN	[82]
	Obtain training data from traditional ILP (Integer Linear Programming) to train an E-CNN	[99]
	Resolve a NP-hard MINLP (Mix-Integer Nonlinear Programming) problem using MADRL	[60]
Distributed deep learning	Beam forming by DRL-based Federal Learning, to reduce communication overhead and avoid phase synchronization	[64]
	Use muti-agent DRL (MADRL) to efficiently coordinate multi-AP and multi-IRS in a distributed manner, at lower information exchange costs	[60]
	DRL trained by MADDPG to tradeoff between energy efficiency and accuracy	[77]
	FL (distributed DRL) to cope with the dynamic context of 6G	[97]
	MADDPG to integrate computation and communication into edge nodes of deep edge network, aiming at pervasive intelligence in edge computing	[98]

## Data Availability

Data availability is not applicable to this article as no new data were created or analyzed in this study.
